# Docetaxel induced severe palmar plantar erythrodysesthesia

**DOI:** 10.11604/pamj.2018.30.70.14741

**Published:** 2018-05-29

**Authors:** Saoussane Kharmoum, Hassan Errihani

**Affiliations:** 1 University Mohamed V-Suissi Rabat, Faculty of Medicine and Pharmacy of Rabat, Department of Medical Oncology, Regional Center of Oncology, Tangier, Morocco; 2 University Mohamed V-Suissi Rabat, Faculty of Medicine and Pharmacy of Rabat, Department of Medical Oncology, National Institute of Oncology, Rabat, Morocco

**Keywords:** Docetaxel, palmar-plantar, erythrodysesthesia

## Images in Medicine

Docetaxel is an important chemotherapeutic agent used alone or in combination for the treatment of many solid tumors, it belongs to the taxane family and is known to have some side effects. Although palmar-plantar erythrodysesthesia (PPE) or hand-foot syndrome is a relatively common toxicity of many anticancer drugs (5-fluorouracil, capecitabine and liposomal doxorubicin), it occurs rarely with docetaxel. We report the case of a 43-year-old North African woman diagnosed with locally advanced breast cancer, received neoadjuvant chemotherapy with three cycles of FEC100 regimen: 5-fluorouracil (500 mg/m^2^), epirubicin (100 mg/m^2^) and cyclophosphamide (500 mg/m^2^). This combination was well tolerated. Then, chemotherapy was switched to docetaxel 100mg/m^²^. Three days after the first cycle, the patient developed an important painful erythema in her two feet, followed by a large desquamation and swelling, which interfered with her functional activities. This reaction was classified as grade 3 PPE according to the National Cancer Institute grading system, and was treated conservatively with emollients and analgesics. In agreement with the patient, the docetaxel chemotherapy was continued with dose reduction of 20%. Symptoms disappeared within 2 weeks following dose reduction.

**Figure 1:**
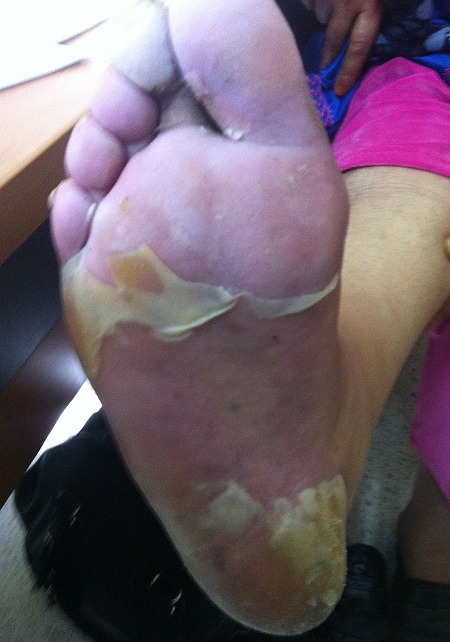
Erythema with a large desquamation and swelling of the foot following docetaxel

